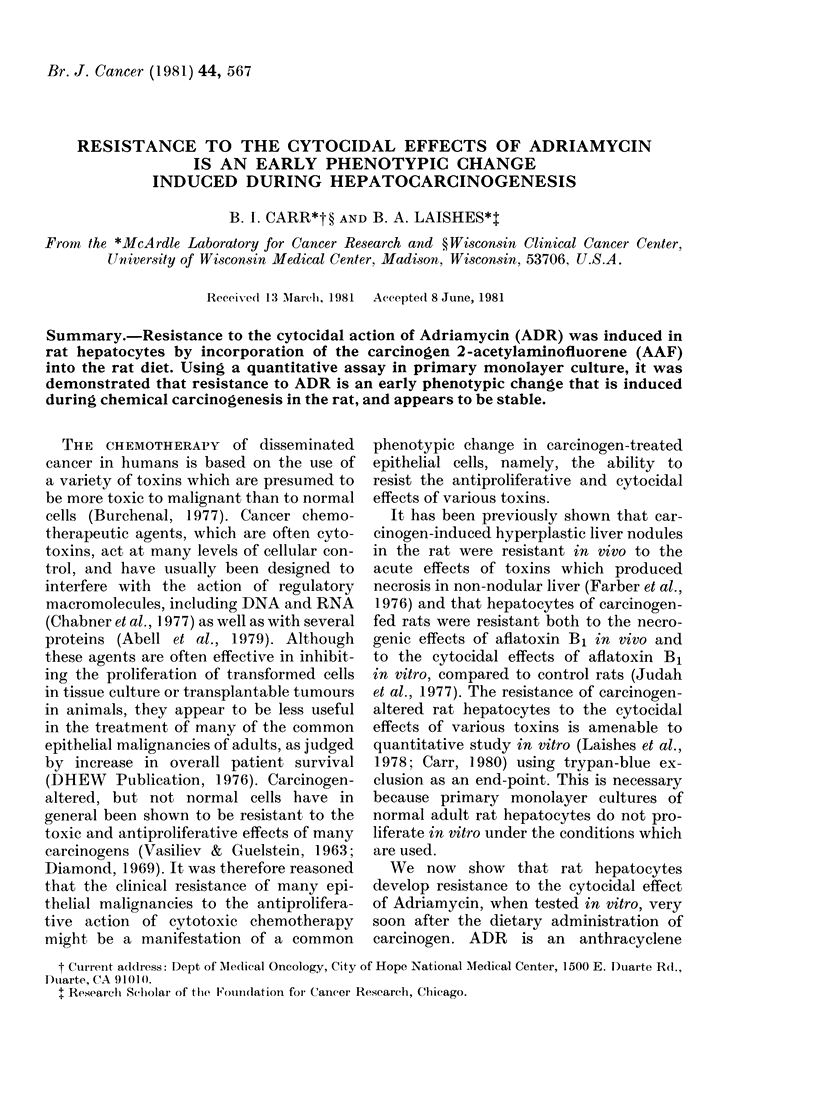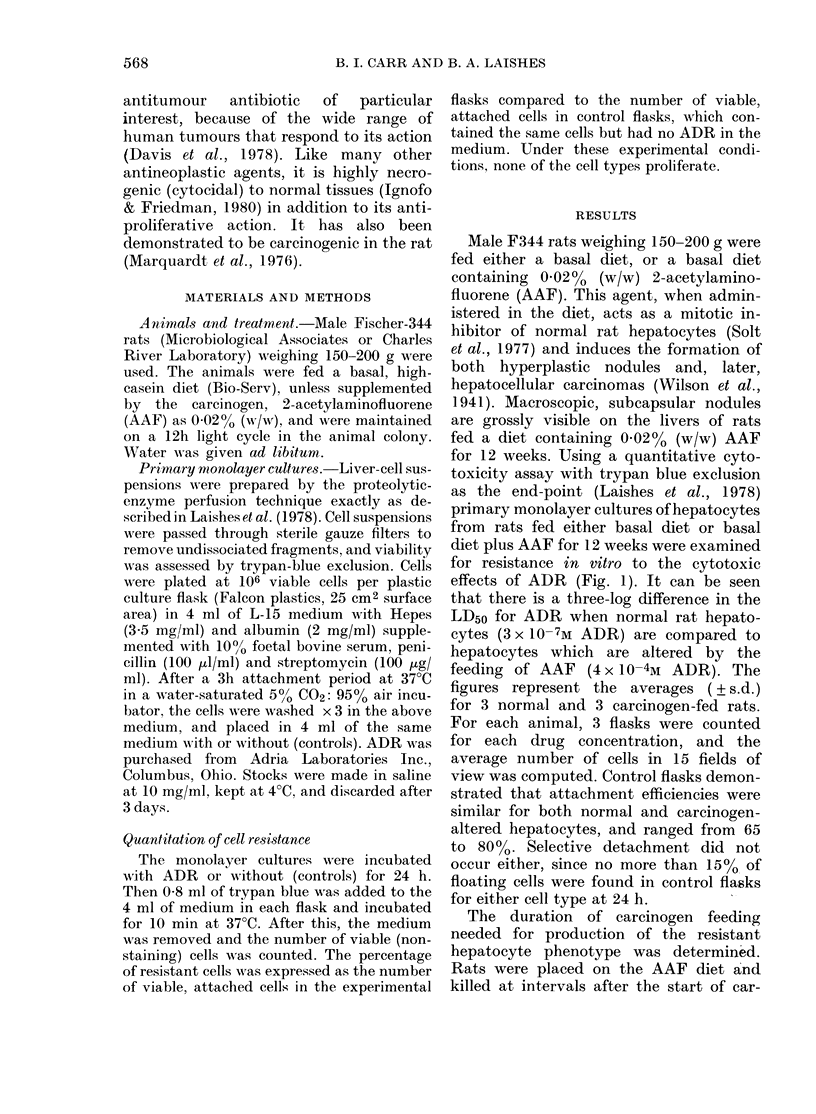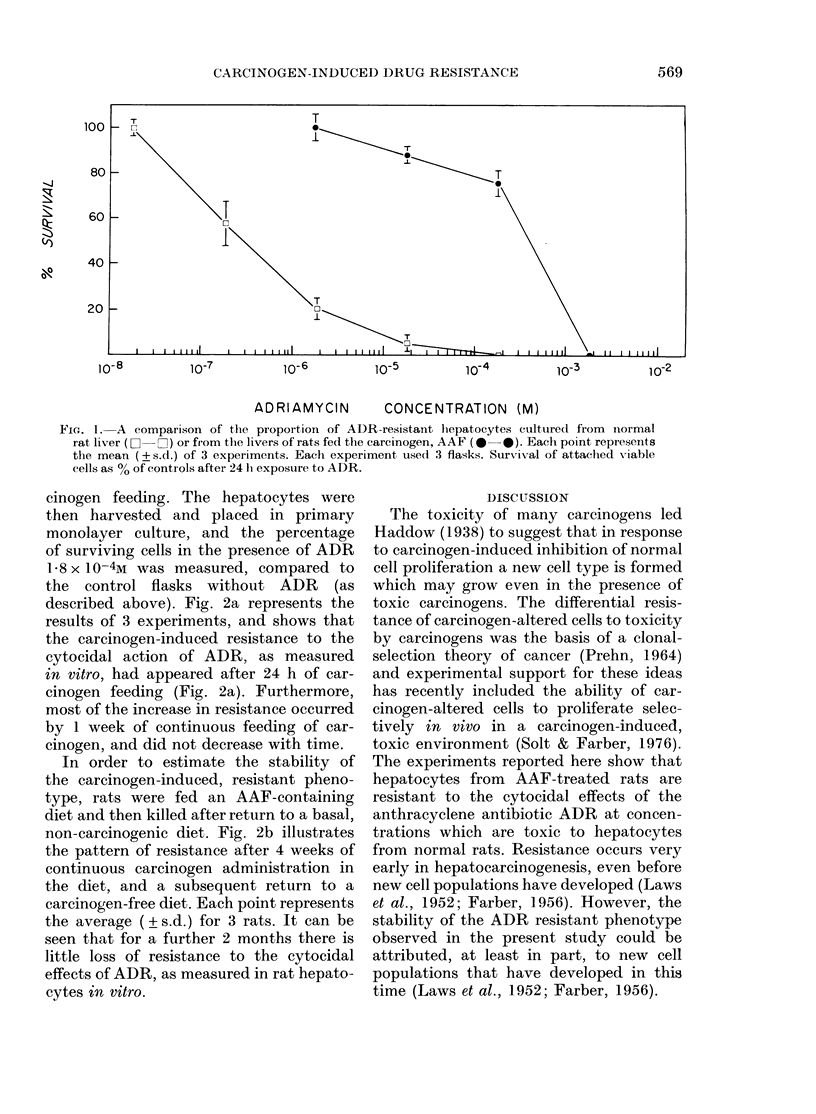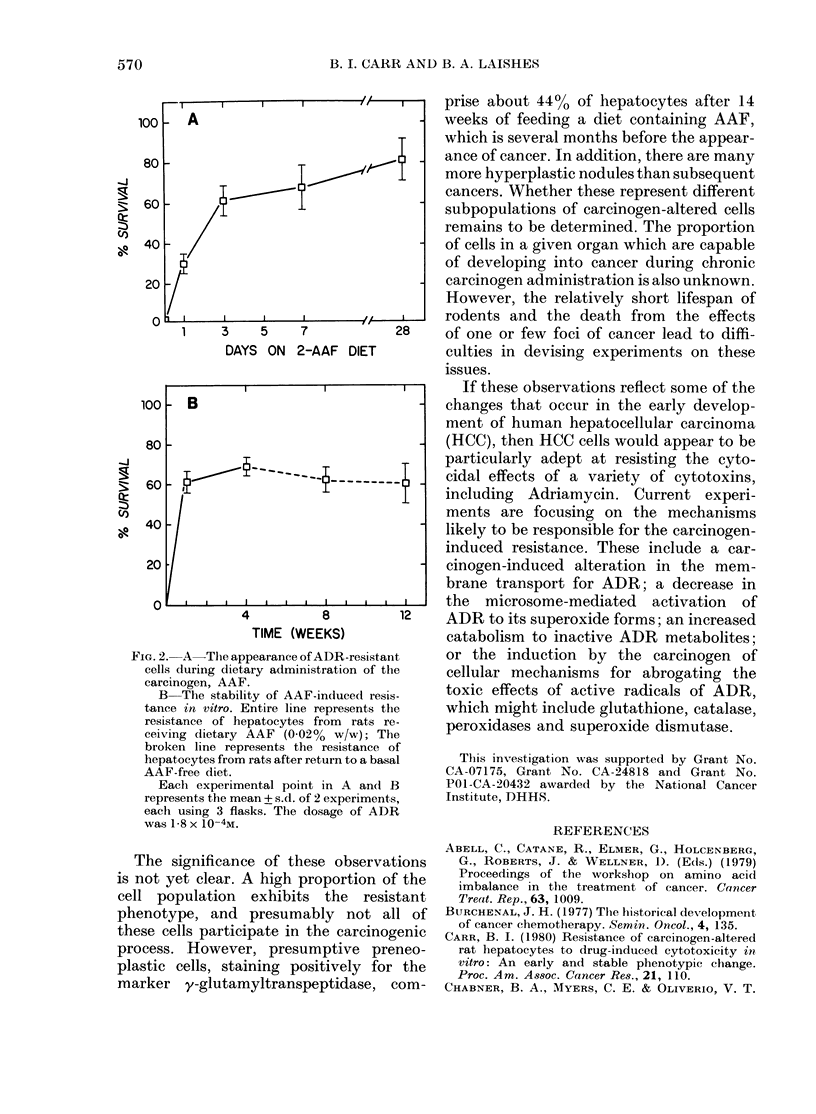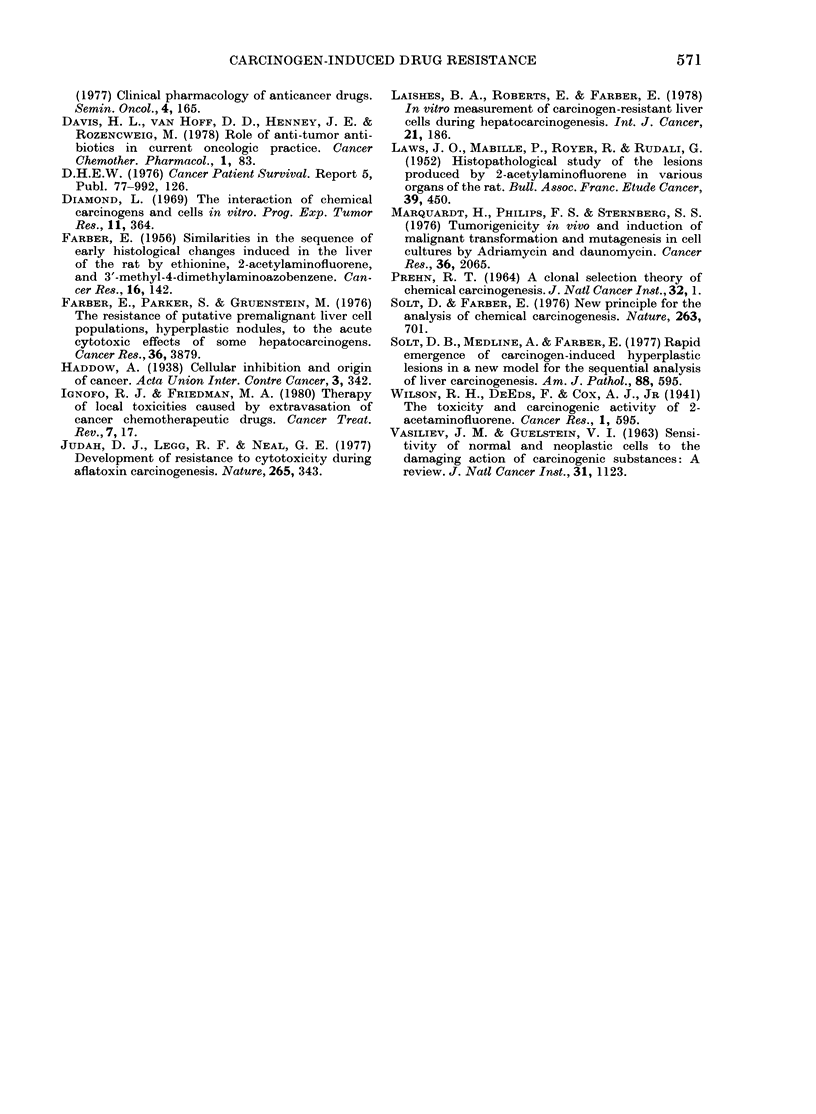# Resistance to the cytocidal effects of adriamycin is an early phenotypic change induced during hepatocarcinogenesis.

**DOI:** 10.1038/bjc.1981.227

**Published:** 1981-10

**Authors:** B. I. Carr, B. A. Laishes

## Abstract

Resistance to the cytocidal action of Adriamycin (ADR) was induced in rat hepatocytes by incorporation of the carcinogen 2-acetylaminofluorene (AAF) into the rat diet. Using a quantitative assay in primary monolayer culture, it was demonstrated that resistance to ADR is an early phenotypic change that is induced during chemical carcinogenesis in the rat, and appears to be stable.


*Br. J. Cancer* (1981) **44**, 567

### RESISTANCE TO THE CYTOCIDAL EFFECTS OF ADRIAMYCIN IS AN EARLY PHENOTYPIC CHANGE INDUCED DURING HEPATOCARCINOGENESIS

B. I. CARR*†§ AND B. A. LAISHES*‡

*From the *McArdle Laboratory for Cancer Research and §Wisconsin Clinical Cancer Center, University of Wisconsin Medical Center, Madison, Wisconsin, 53706, U.S.A.*

Received 13 March, 1981 Accepted 8 June, 1981

**Summary.—Resistance to the cytocidal action of Adriamycin (ADR) was induced in rat hepatocytes by incorporation of the carcinogen 2-acetylaminofluorene (AAF) into the rat diet. Using a quantitative assay in primary monolayer culture, it was demonstrated that resistance to ADR is an early phenotypic change that is induced during chemical carcinogenesis in the rat, and appears to be stable.**

THE CHEMOTHERAPY of disseminated cancer in humans is based on the use of a variety of toxins which are presumed to be more toxic to malignant than to normal cells (Burchenal, 1977). Cancer chemotherapeutic agents, which are often cytotoxins, act at many levels of cellular control, and have usually been designed to interfere with the action of regulatory macromolecules, including DNA and RNA (Chabner *et al.*, 1977) as well as with several proteins (Abell *et al.*, 1979). Although these agents are often effective in inhibiting the proliferation of transformed cells in tissue culture or transplantable tumours in animals, they appear to be less useful in the treatment of many of the common epithelial malignancies of adults, as judged by increase in overall patient survival (DHEW Publication, 1976). Carcinogen-altered, but not normal cells have in general been shown to be resistant to the toxic and antiproliferative effects of many carcinogens (Vasiliev & Guelstein, 1963; Diamond, 1969). It was therefore reasoned that the clinical resistance of many epithelial malignancies to the antiproliferative action of cytotoxic chemotherapy might be a manifestation of a common phenotypic change in carcinogen-treated epithelial cells, namely, the ability to resist the antiproliferative and cytocidal effects of various toxins.

It has been previously shown that carcinogen-induced hyperplastic liver nodules in the rat were resistant *in vivo* to the acute effects of toxins which produced necrosis in non-nodular liver (Farber *et al.*, 1976) and that hepatocytes of carcinogen-fed rats were resistant both to the necrogenic effects of aflatoxin $B_1$ *in vivo* and to the cytocidal effects of aflatoxin $B_1$ *in vitro*, compared to control rats (Judah *et al.*, 1977). The resistance of carcinogen-altered rat hepatocytes to the cytocidal effects of various toxins is amenable to quantitative study *in vitro* (Laishes *et al.*, 1978; Carr, 1980) using trypan-blue exclusion as an end-point. This is necessary because primary monolayer cultures of normal adult rat hepatocytes do not proliferate *in vitro* under the conditions which are used.

We now show that rat hepatocytes develop resistance to the cytocidal effect of Adriamycin, when tested *in vitro*, very soon after the dietary administration of carcinogen. ADR is an anthracyclene

† Current address: Dept of Medical Oncology, City of Hope National Medical Center, 1500 E. Duarte Rd., Duarte, CA 91010.

‡ Research Scholar of the Foundation for Cancer Research, Chicago.

antitumour antibiotic of particular interest, because of the wide range of human tumours that respond to its action (Davis *et al.*, 1978). Like many other antineoplastic agents, it is highly necrogenic (cytocidal) to normal tissues (Ignofo & Friedman, 1980) in addition to its antiproliferative action. It has also been demonstrated to be carcinogenic in the rat (Marquardt *et al.*, 1976).

### MATERIALS AND METHODS

*Animals and treatment.*—Male Fischer-344 rats (Microbiological Associates or Charles River Laboratory) weighing 150–200 g were used. The animals were fed a basal, high-casein diet (Bio-Serv), unless supplemented by the carcinogen, 2-acetylaminofluorene (AAF) as 0·02% (w/w), and were maintained on a 12h light cycle in the animal colony. Water was given *ad libitum*.

*Primary monolayer cultures.*—Liver-cell suspensions were prepared by the proteolytic-enzyme perfusion technique exactly as described in Laishes *et al.* (1978). Cell suspensions were passed through sterile gauze filters to remove undissociated fragments, and viability was assessed by trypan-blue exclusion. Cells were plated at $10^6$ viable cells per plastic culture flask (Falcon plastics, 25 $cm^2$ surface area) in 4 ml of L-15 medium with Hepes (3·5 mg/ml) and albumin (2 mg/ml) supplemented with 10% foetal bovine serum, penicillin (100 μl/ml) and streptomycin (100 μg/ml). After a 3h attachment period at 37°C in a water-saturated 5% $CO_2$: 95% air incubator, the cells were washed ×3 in the above medium, and placed in 4 ml of the same medium with or without (controls). ADR was purchased from Adria Laboratories Inc., Columbus, Ohio. Stocks were made in saline at 10 mg/ml, kept at 4°C, and discarded after 3 days.

### *Quantitation of cell resistance*

The monolayer cultures were incubated with ADR or without (controls) for 24 h. Then 0·8 ml of trypan blue was added to the 4 ml of medium in each flask and incubated for 10 min at 37°C. After this, the medium was removed and the number of viable (non-staining) cells was counted. The percentage of resistant cells was expressed as the number of viable, attached cells in the experimental flasks compared to the number of viable, attached cells in control flasks, which contained the same cells but had no ADR in the medium. Under these experimental conditions, none of the cell types proliferate.

### RESULTS

Male F344 rats weighing 150–200 g were fed either a basal diet, or a basal diet containing 0·02% (w/w) 2-acetylaminofluorene (AAF). This agent, when administered in the diet, acts as a mitotic inhibitor of normal rat hepatocytes (Solt *et al.*, 1977) and induces the formation of both hyperplastic nodules and, later, hepatocellular carcinomas (Wilson *et al.*, 1941). Macroscopic, subcapsular nodules are grossly visible on the livers of rats fed a diet containing 0·02% (w/w) AAF for 12 weeks. Using a quantitative cytotoxicity assay with trypan blue exclusion as the end-point (Laishes *et al.*, 1978) primary monolayer cultures of hepatocytes from rats fed either basal diet or basal diet plus AAF for 12 weeks were examined for resistance *in vitro* to the cytotoxic effects of ADR (Fig. 1). It can be seen that there is a three-log difference in the $LD_{50}$ for ADR when normal rat hepatocytes ($3\times10^{-7}$M ADR) are compared to hepatocytes which are altered by the feeding of AAF ($4\times10^{-4}$M ADR). The figures represent the averages (±s.d.) for 3 normal and 3 carcinogen-fed rats. For each animal, 3 flasks were counted for each drug concentration, and the average number of cells in 15 fields of view was computed. Control flasks demonstrated that attachment efficiencies were similar for both normal and carcinogen-altered hepatocytes, and ranged from 65 to 80%. Selective detachment did not occur either, since no more than 15% of floating cells were found in control flasks for either cell type at 24 h.

The duration of carcinogen feeding needed for production of the resistant hepatocyte phenotype was determined. Rats were placed on the AAF diet and killed at intervals after the start of car-

FIG. 1.—A comparison of the proportion of ADR-resistant hepatocytes cultured from normal rat liver (□—□) or from the livers of rats fed the carcinogen, AAF (●—●). Each point represents the mean (±s.d.) of 3 experiments. Each experiment used 3 flasks. Survival of attached viable cells as % of controls after 24 h exposure to ADR.

cinogen feeding. The hepatocytes were then harvested and placed in primary monolayer culture, and the percentage of surviving cells in the presence of ADR $1 \cdot 8 \times 10^{-4}$M was measured, compared to the control flasks without ADR (as described above). Fig. 2a represents the results of 3 experiments, and shows that the carcinogen-induced resistance to the cytocidal action of ADR, as measured *in vitro*, had appeared after 24 h of carcinogen feeding (Fig. 2a). Furthermore, most of the increase in resistance occurred by 1 week of continuous feeding of carcinogen, and did not decrease with time.

In order to estimate the stability of the carcinogen-induced, resistant phenotype, rats were fed an AAF-containing diet and then killed after return to a basal, non-carcinogenic diet. Fig. 2b illustrates the pattern of resistance after 4 weeks of continuous carcinogen administration in the diet, and a subsequent return to a carcinogen-free diet. Each point represents the average (±s.d.) for 3 rats. It can be seen that for a further 2 months there is little loss of resistance to the cytocidal effects of ADR, as measured in rat hepatocytes *in vitro*.

### DISCUSSION

The toxicity of many carcinogens led Haddow (1938) to suggest that in response to carcinogen-induced inhibition of normal cell proliferation a new cell type is formed which may grow even in the presence of toxic carcinogens. The differential resistance of carcinogen-altered cells to toxicity by carcinogens was the basis of a clonal-selection theory of cancer (Prehn, 1964) and experimental support for these ideas has recently included the ability of carcinogen-altered cells to proliferate selectively *in vivo* in a carcinogen-induced, toxic environment (Solt & Farber, 1976). The experiments reported here show that hepatocytes from AAF-treated rats are resistant to the cytocidal effects of the anthracyclene antibiotic ADR at concentrations which are toxic to hepatocytes from normal rats. Resistance occurs very early in hepatocarcinogenesis, even before new cell populations have developed (Laws *et al.*, 1952; Farber, 1956). However, the stability of the ADR resistant phenotype observed in the present study could be attributed, at least in part, to new cell populations that have developed in this time (Laws *et al.*, 1952; Farber, 1956).

FIG. 2.—A—The appearance of ADR-resistant cells during dietary administration of the carcinogen, AAF.

B—The stability of AAF-induced resistance *in vitro*. Entire line represents the resistance of hepatocytes from rats receiving dietary AAF (0·02% w/w); The broken line represents the resistance of hepatocytes from rats after return to a basal AAF-free diet.

Each experimental point in A and B represents the mean $\pm$ s.d. of 2 experiments, each using 3 flasks. The dosage of ADR was $1 \cdot 8 \times 10^{-4}$M.

The significance of these observations is not yet clear. A high proportion of the cell population exhibits the resistant phenotype, and presumably not all of these cells participate in the carcinogenic process. However, presumptive preneoplastic cells, staining positively for the marker $\gamma$-glutamyltranspeptidase, comprise about 44% of hepatocytes after 14 weeks of feeding a diet containing AAF, which is several months before the appearance of cancer. In addition, there are many more hyperplastic nodules than subsequent cancers. Whether these represent different subpopulations of carcinogen-altered cells remains to be determined. The proportion of cells in a given organ which are capable of developing into cancer during chronic carcinogen administration is also unknown. However, the relatively short lifespan of rodents and the death from the effects of one or few foci of cancer lead to difficulties in devising experiments on these issues.

If these observations reflect some of the changes that occur in the early development of human hepatocellular carcinoma (HCC), then HCC cells would appear to be particularly adept at resisting the cytocidal effects of a variety of cytotoxins, including Adriamycin. Current experiments are focusing on the mechanisms likely to be responsible for the carcinogen-induced resistance. These include a carcinogen-induced alteration in the membrane transport for ADR; a decrease in the microsome-mediated activation of ADR to its superoxide forms; an increased catabolism to inactive ADR metabolites; or the induction by the carcinogen of cellular mechanisms for abrogating the toxic effects of active radicals of ADR, which might include glutathione, catalase, peroxidases and superoxide dismutase.

This investigation was supported by Grant No. CA-07175, Grant No. CA-24818 and Grant No. P01-CA-20432 awarded by the National Cancer Institute, DHHS.

### REFERENCES

ABELL, C., CATANE, R., ELMER, G., HOLCENBERG, G., ROBERTS, J. & WELLNER, D. (Eds.) (1979) Proceedings of the workshop on amino acid imbalance in the treatment of cancer. *Cancer Treat. Rep.*, **63**, 1009.

BURCHENAL, J. H. (1977) The historical development of cancer chemotherapy. *Semin. Oncol.*, **4**, 135.

CARR, B. I. (1980) Resistance of carcinogen-altered rat hepatocytes to drug-induced cytotoxicity *in vitro*: An early and stable phenotypic change. *Proc. Am. Assoc. Cancer Res.*, **21**, 110.

CHABNER, B. A., MYERS, C. E. & OLIVERIO, V. T.

(1977) Clinical pharmacology of anticancer drugs. *Semin. Oncol.*, **4**, 165.

Davis, H. L., van Hoff, D. D., Henney, J. E. & Rozencweig, M. (1978) Role of anti-tumor antibiotics in current oncologic practice. *Cancer Chemother. Pharmacol.*, **1**, 83.

D.H.E.W. (1976) *Cancer Patient Survival.* Report 5, Publ. 77–992, 126.

Diamond, L. (1969) The interaction of chemical carcinogens and cells *in vitro*. *Prog. Exp. Tumor Res.*, **11**, 364.

Farber, E. (1956) Similarities in the sequence of early histological changes induced in the liver of the rat by ethionine, 2-acetylaminofluorene, and 3′-methyl-4-dimethylaminoazobenzene. *Cancer Res.*, **16**, 142.

Farber, E., Parker, S. & Gruenstein, M. (1976) The resistance of putative premalignant liver cell populations, hyperplastic nodules, to the acute cytotoxic effects of some hepatocarcinogens. *Cancer Res.*, **36**, 3879.

Haddow, A. (1938) Cellular inhibition and origin of cancer. *Acta Union Inter. Contre Cancer*, **3**, 342.

Ignofo, R. J. & Friedman, M. A. (1980) Therapy of local toxicities caused by extravasation of cancer chemotherapeutic drugs. *Cancer Treat. Rev.*, **7**, 17.

Judah, D. J., Legg, R. F. & Neal, G. E. (1977) Development of resistance to cytotoxicity during aflatoxin carcinogenesis. *Nature*, **265**, 343.

Laishes, B. A., Roberts, E. & Farber, E. (1978) *In vitro* measurement of carcinogen-resistant liver cells during hepatocarcinogenesis. *Int. J. Cancer*, **21**, 186.

Laws, J. O., Mabille, P., Royer, R. & Rudali, G. (1952) Histopathological study of the lesions produced by 2-acetylaminofluorene in various organs of the rat. *Bull. Assoc. Franc. Etude Cancer*, **39**, 450.

Marquardt, H., Philips, F. S. & Sternberg, S. S. (1976) Tumorigenicity *in vivo* and induction of malignant transformation and mutagenesis in cell cultures by Adriamycin and daunomycin. *Cancer Res.*, **36**, 2065.

Prehn, R. T. (1964) A clonal selection theory of chemical carcinogenesis. *J. Natl Cancer Inst.*, **32**, 1.

Solt, D. & Farber, E. (1976) New principle for the analysis of chemical carcinogenesis. *Nature*, **263**, 701.

Solt, D. B., Medline, A. & Farber, E. (1977) Rapid emergence of carcinogen-induced hyperplastic lesions in a new model for the sequential analysis of liver carcinogenesis. *Am. J. Pathol.*, **88**, 595.

Wilson, R. H., DeEds, F. & Cox, A. J., Jr (1941) The toxicity and carcinogenic activity of 2-acetaminofluorene. *Cancer Res.*, **1**, 595.

Vasiliev, J. M. & Guelstein, V. I. (1963) Sensitivity of normal and neoplastic cells to the damaging action of carcinogenic substances: A review. *J. Natl Cancer Inst.*, **31**, 1123.